# Fine Mapping of *qAL5.2* Controlling Anther Length in *Oryza sativa*

**DOI:** 10.3390/plants13081130

**Published:** 2024-04-18

**Authors:** Xinyong Liu, Zixuan Yu, Xiaohong Tong, Longxue Chang, Jie Huang, Yifeng Wang, Jiezheng Ying, Xingwang Li, Shen Ni, Jian Zhang

**Affiliations:** 1 State Key Laboratory of Rice Biology and Breeding, China National Rice Research Institute, Hangzhou 311400, China; liuxinyong1234@gmail.com (X.L.); wangyifeng@caas.cn (Y.W.);; 2 College of Life Science and Technology, Huazhong Agricultural University, Wuhan 430070, China

**Keywords:** anther length, outcrossing, *Oryza sativa*, fine mapping, hybrid rice

## Abstract

Anther length is the critical floral trait determining hybrid rice seed production and is controlled by many quantitative trait loci (QTL). However, the cloning of genes specifically controlling anther size has yet to be reported. Here, we report the fine mapping of *qAL5.2* for anther size using backcross inbred lines (BILs) in the genetic background of *Oryza sativa indica* Huazhan (HZ). Gene chip analysis on the BC_4_F_2_ and BC_5_F_1_ population identified effective loci on Chr1, Chr5, and Chr8 and two genomic regions on Chr5, named *qAL5.1* and *qAL5.2*. *qAL5.2* was identified in both populations with LOD values of 17.54 and 10.19, which explained 35.73% and 25.1% of the phenotypic variances, respectively. Ultimately *qAL5.2* was localized to a 73 kb region between HK139 and HK140 on chromosome 5. And we constructed two near-isogenic lines (NILs) for RNA-seq analysis, named NIL-qAL5.2^HZ^ and NIL-qAL5.2^KLY^, respectively. The result of the GO enrichment analysis revealed that differential genes were significantly enriched in the carbohydrate metabolic process, extracellular region, and nucleic acid binding transcription, and KEGG enrichment analysis revealed that alpha-linolenic acid metabolism was significantly enriched. Meanwhile, candidate genes of *qAL5.2* were analyzed in RNA-seq, and it was found that *ORF8* is differentially expressed between NIL-qAL5.2^HZ^ and NIL-qAL5.2^KLY^. The fine mapping of *qAL5.2* conferring anther length will promote the breed improvement of the restorer line and understanding of the mechanisms driving crop mating patterns.

## 1. Introduction

Rice is one of the most populous crops in the world, and hybrid rice technology is an important technique for improving rice yield. Hybrid rice technology breaks the limitations of self-pollinating crops, using outcrossing pollination between restoring and sterile lines to obtain offspring and exhibiting strong heterosis in F1 generation, which can significantly improve rice yield [1]. Hybrid rice technology mainly includes the “three-line system” and the “two-line system”. The earliest hybrid varieties were obtained through the three-line system. Hybrid varieties developed with a three-line (CMS, maintainer, and restorer) system can improve 20–40% higher yields and are widely recommended since 1976 in China [1].

Three-line hybrid technology includes rice cytoplasmic male sterile lines, a rice cytoplasmic male sterile maintainer line, and a rice cytoplasmic male sterile restorer line [2,3]. *WA352* and *Rf4* were the first genes to be cloned from wild abortive cytoplasmic male sterility (CMS-WA) [4,5]. It consists of two main steps, first breeding the female sterile parent by crossing the sterile line with the maintainer line and then crossing the sterile line with the restorer line to produce the F1 hybrid seed. In subsequent research, scientists discovered the photoperiod/thermo-sensitive male sterile lines and achieved “two-line system” technology without the maintainer line, which effectively simplified the process of hybrid rice seed production [6,7]. Since rice is a self-pollinating crop and needs to be pollinated artificially, enough restorer lines should be planted to ensure that the sterile line can obtain sufficient pollen to complete fertilization, whether the three-line system or the two-line system [8]. However, the overplanting of restorer lines will reduce the area of sterile lines, causing a decrease in the yield of hybrid seed production. In recent years, the high labor cost and low yield in hybrid rice seed production led to high seed prices, severely limiting the promotion and application of hybrid rice [9,10].

Improving the anther size of the restoring line or the exsertion rate of the sterile line stigma can effectively increase the yield of hybrid seed production [11]. Some researchers have detected QTL for anther length by different populations. Tazib et al. detected four major QTL located on chromosomes 2, 3, 5, and 7 through backcross inbred lines derived from the backcrossing of the rice cultivars (Nipponbare × Kasalath) × Nipponbare [12]. Four QTL for anther length were also detected on chromosomes 2, 3, and 8 by populations derived from a cross between an indica (SPR1) and a common wild rice [13]. Recently, some new anther QTL were identified by the advanced backcross line of *O. longistaminata* accession W1508 and chromosomal segment substitution lines in the genetic background of *O. sativa* Taichung 65 and found anther size was regulated by cell elongation and cell proliferation in two different ways [14]. However, the region of these QTL is too rough for accurate mapping, making it challenging to apply in hybrid seed production.

In this study, we detected six QTL by backcross inbred lines derived from two rice cultivars HZ and Koliya (KLY). And *qAL5.2* was narrowed down to a 73 kb region by fine mapping. Regulatory pathways of anther size were analyzed by RNA-seq, and it was revealed that anther size might be regulated by pathways related to alpha-linolenic acid metabolism.

## 2. Results

### 2.1. Anther Morphology of NIL-HZ and NIL-KLY

**Figure 1:**
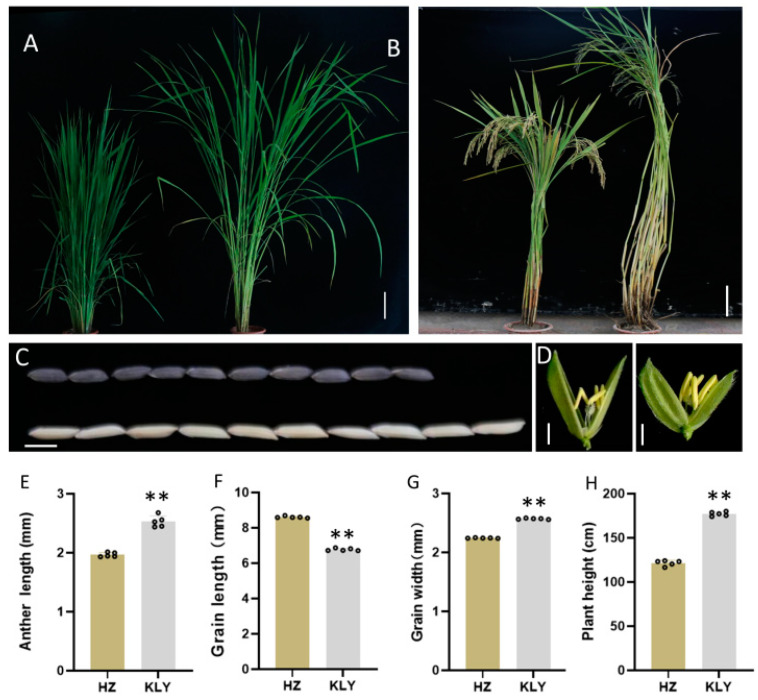
The phenotypic characterization of Huazhan (HZ) and Koliya (KLY). (**A**,**B**) The gestational stage (**A**) and maturation stage (**B**) of HZ and KLY grown under natural field conditions, Bar = 25 cm. (**C**) The grain length of HZ (down) and KLY (up), Bar = 5 cm. (**D**) The anther of the spikelet of HZ (left) and KLY (right), Bar = 1 cm. (**E**–**H**) The statistics of the agronomic traits of HZ and KLY: anther length (**E**), grain length (**F**), grain width (**G**), and plant height (**H**). Values are means ± SD from three biological replicates. Asterisks indicate statistical significance as determined by Student’s *t*-test (** *p* < 0.01).

**Figure 2:**
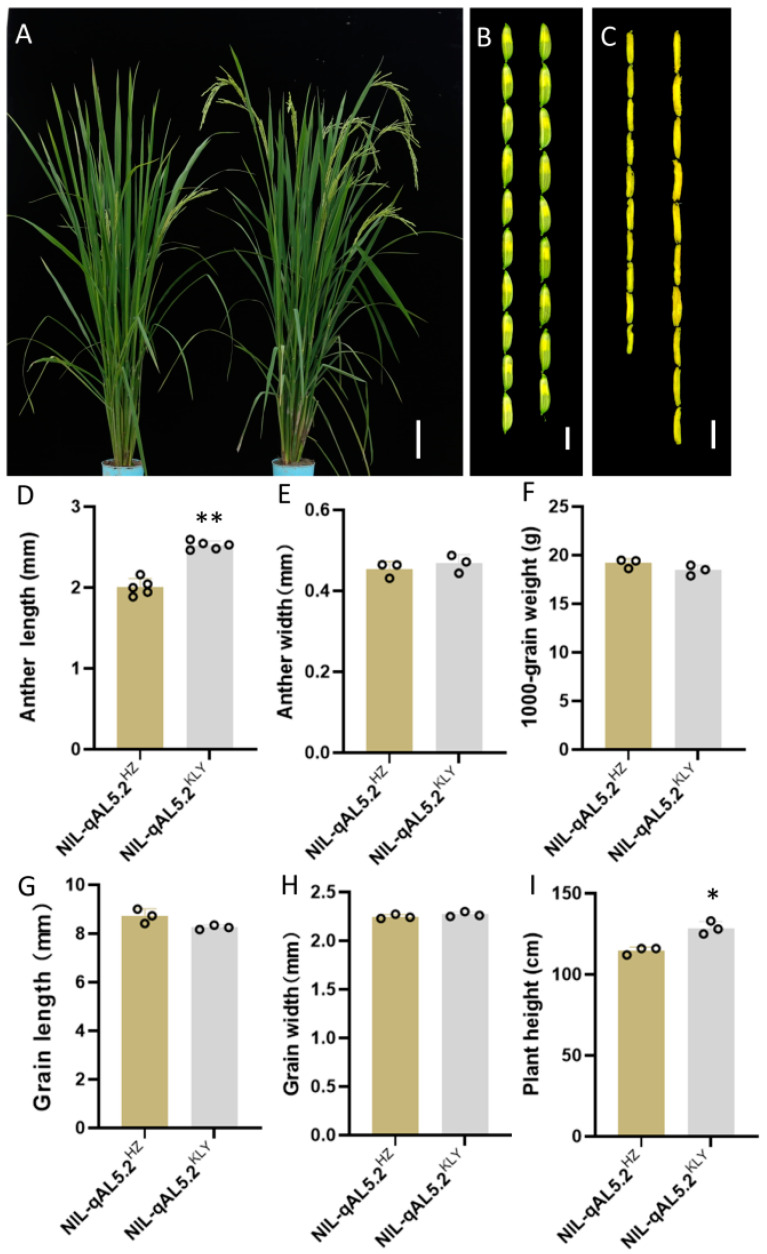
The phenotypic characterization of NIL-HZ and NIL-KLY. (**A**) The gestational stage of NIL-HZ and NIL-KLY grown under natural field conditions, Bar = 10 cm. (**B**,**C**) The length of the spikelet (**B**) and anther (**C**) of NIL-HZ (left) and NIL-KLY (right), Bar = 5 mm and 2 mm. (**D**–**I**) The statistics of the agronomic traits of NIL-HZ and NIL-KLY: anther length (**D**), anther width (**E**), the weight of 1000 grains (**F**), grain length (**G**), grain width (**H**), and plant height (**I**). Values are means ± SD from three biological replicates. Asterisks indicate statistical significance as determined by Student’s *t*-test (* *p* < 0.05; ** *p* < 0.01).

To discover probable quantitative trait loci (QTL) controlling anther length, we investigated the parental phenotype of the restore line Huazhan (HZ, *Oryza sativa* ssp. *Indica*) and Koliya (KLY) from south-east Asia (Figure 1). The anther length of HZ and KLY were 1.96 ± 0.04 mm and 2.53 ± 0.09 mm, respectively. However, KLY exhibits much shorter grain length than HZ (Figure 1F). The grain length of KLY was only 6.5 mm, which is 23% shorter than that of HZ, resulting in a much higher anther/glume length ratio (Figure 1E,F). Subsequently, we constructed a mapping population and the near-isogenic line (NIL) using anther length as the trait for selection (Figure S1). At the same time, we investigated the phenotype of NIL-HZ and NIL-KLY derived from BC_4_F_1_ with the genetic background of HZ (Figure 2). The anther length of NIL-HZ and NIL-KLY were 2.00 ± 0.1 mm and 2.52 ± 0.05 mm, respectively (Figure 2C,D). At the same time, there were no noticeable significant differences in anther width, 1000-grain weight, grain length, or grain width but there were in plant height (Figure 2E–I).

### 2.2. Effects of qAL5.2 for Anther Length

**Figure 3:**
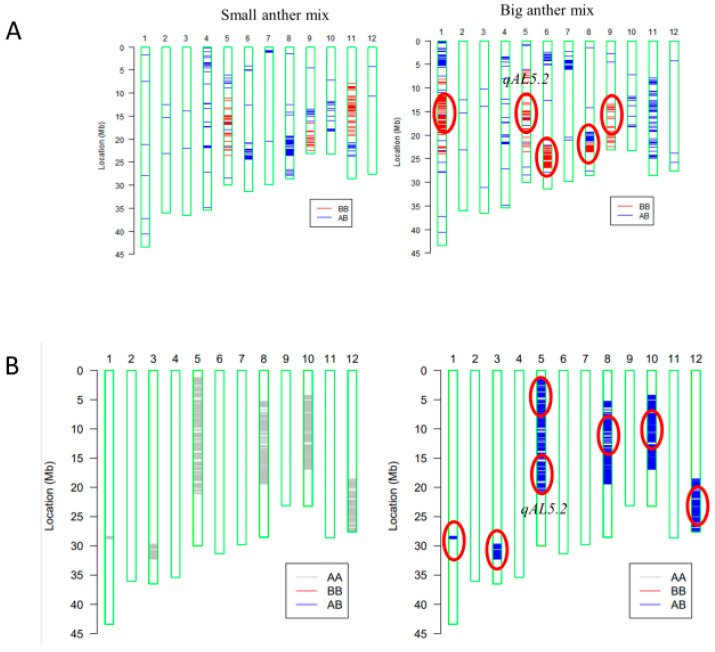
Primary mapping of QTL for anther length using gene chips containing 10 K molecular markers. (**A**) Analysis of plants with extremely short and long anther size in BC_4_F_2_ (n = 20). (**B**) Analysis in F1 of BC_5_F_1_ (n = 10). AA stands for HZ; BB stands for KLY. Red circles represent QTL locations.

For mapping quantitative trait loci (QTL) of anther length, the BC_4_F_2_ population was constructed with the genetic background of HZ (Figure S1). BC_4_F_2_ individuals with extremely large or small anthers were pool sampled, respectively, for gene chip analysis (Figure 3A). In the BC_4_F_2_ population (n = 20), five loci on Chr1-1, Chr5, Chr6, Chr8-1, and Chr9 (Figure 3A) were detected. Subsequently, six loci were detected on Chr1-2, Chr3, Chr5, Chr8-2, Chr10, and Chr12 in the BC_5_F_1_ population (n = 10) (Figure 3B).

**Figure 4:**
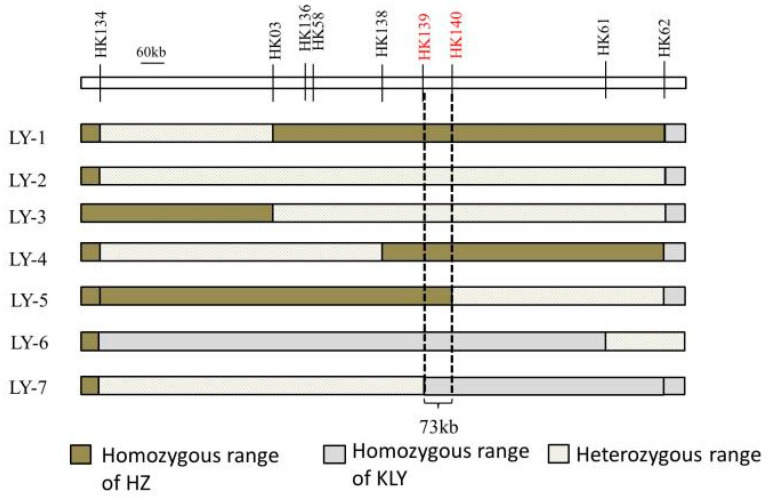
The genotypic compositions of populations in the segregating regions.

The draft mapping results revealed that two genomic regions in Chr5 were repeatedly detected in the BC_4_F_2_ and BC_5_F_1_ populations and thereafter named as *qAL5.1* and *qAL5.2*, respectively. To fine map QTL of anther length, we constructed seven BC_5_F_2_ populations with the segregating regions covering the chip mark R0516540382GA- F0520537816TG in *qAL5.2*, and QTL analysis was performed (Figure 4). The result showed that *qAL5.2* was observed in LY-2 and LY-3 populations. In LY-2 and LY-3 populations, the peak LOD values were 17.54 and 10.19, and the additive effects were 0.12 mm and 0.08 mm, explaining 35.73% and 25.1% of the phenotypic variances, respectively (Figure S2). There were no significant QTL in other populations (Table 1). So *qAL5.2* might be localized to a 73 kb interval between HK139 and HK140 on chromosome 5.

### 2.3. Enrichment Analysis of RNA-Seq

**Figure 5:**
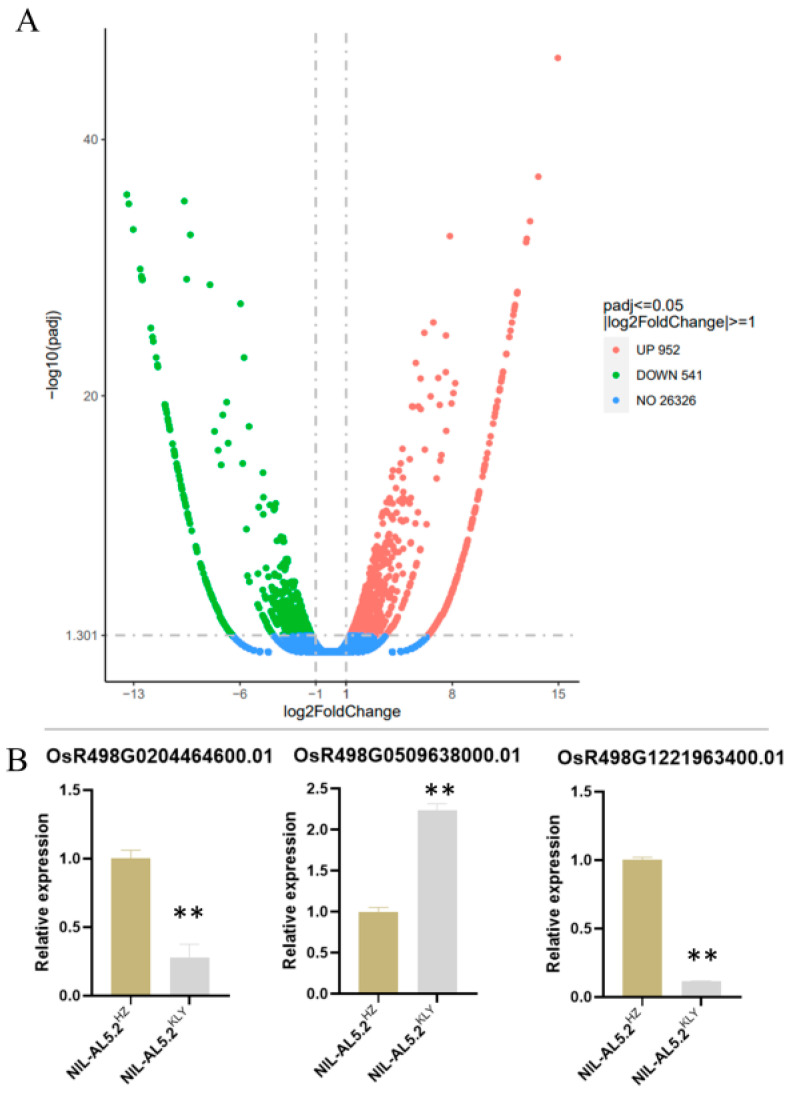
Differential expression analysis in RNA-seq. (**A**) Volcano plot of mRNA expression in S4 anther (pollen microspore metaphase stage). (**B**) Verification of DEG by qRT-PCR. Asterisks indicate statistical significance as determined by Student’s *t*-test (** *p* < 0.01).

To analyze how *qAL5.2* affects the development of the anther, we conducted an RNA-sequence experiment using the anther samples in the S4-stage (pollen microspore metaphase stage) from the near-isogenic lines NIL-qAL5-2^HZ^ and NIL-qAL5-2^KLY^, respectively. Compared with NIL-qAL5-2^KLY^, there were 952 genes observably up-regulated and 541 genes significantly down-regulated in the S4-stage anther in differential expression gene (DEG) analysis (Figure 5A and Table S3). In order to verify the DEG results, three genes were randomly detected by qRT-PCR, and the results showed that the relative expression of the three genes was consistent with the DEG results (Figure 5B). So it could be used in the following analysis.

**Figure 6:**
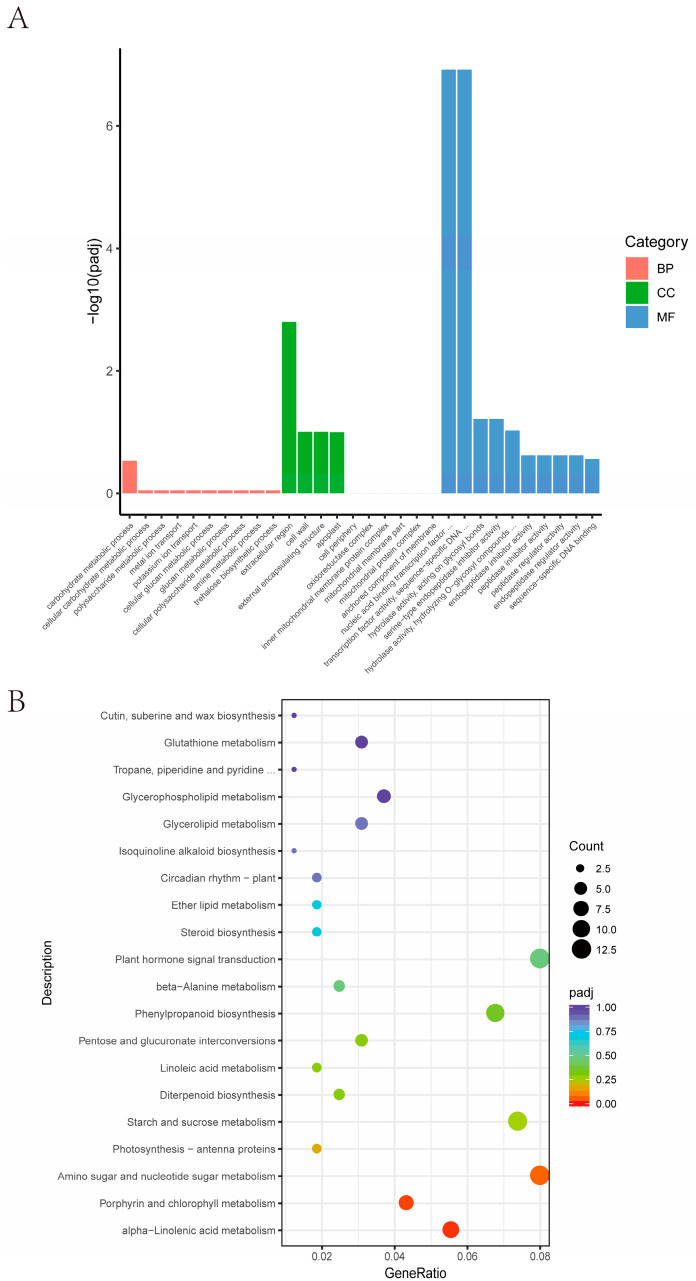
Candidate gene selection from RNA-seq analysis. (**A**) Enrichment of GOs for differentially expressed mRNAs. (**B**) Bubble plot of pathways for differentially expressed mRNAs. BP biological process; CC cellular component; MF molecular function.

The result of the GO enrichment analysis showed that differential genes were mainly enriched in the carbohydrate metabolic process, extracellular region, and nucleic acid binding transcription (Figure 6A). The result of the KEGG enrichment analysis revealed that they were significantly (*p* < 0.05) enriched in alpha-linolenic acid metabolism, porphyrin and chlorophyll metabolism, and amino sugar and nucleotide sugar metabolism pathways (Figure 6B), indicating *qAL5.2* is involved in the regulation of the energy metabolism of anthers.

### 2.4. Candidate Gene Analysis of qAL5.2

There were eight predicted reading frames (ORFs) covered by *qAL5.2*. We analyzed the expression of all eight candidate genes using the data from the Rice Genome Annotation Project. We found that only *ORF1*, *ORF3*, *ORF4*, and *ORF8* showed expression in the rice anther, and *ORF8* had a particularly high level (Table 2 and Figure S3). And only *ORF8* expression has a significant difference in RNA-Seq.

**Figure 7:**
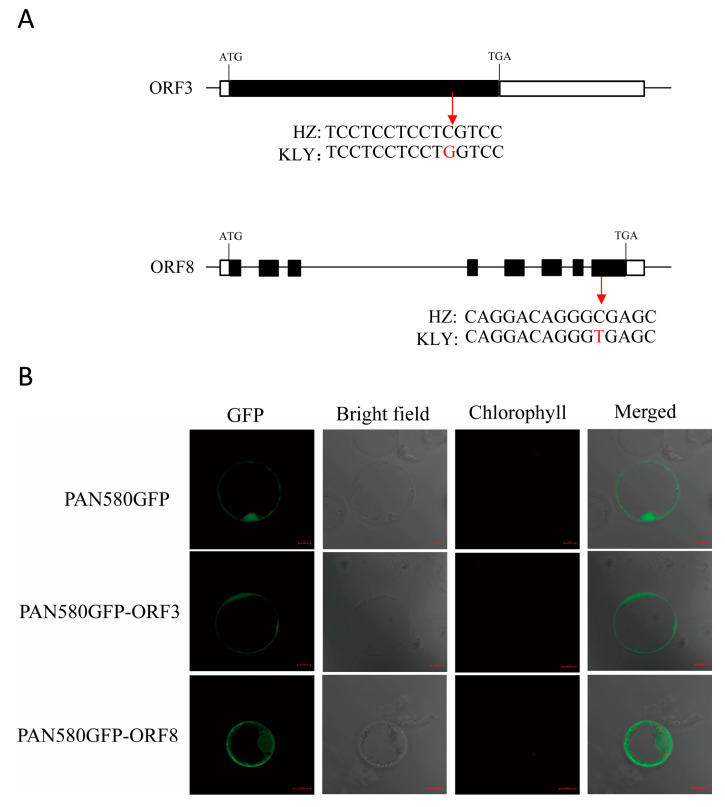
The identification of candidate genes for anther length. (**A**) A schematic diagram of the SNP of the candidate genes between HZ and KLY. An SNP occurred in *ORF3*, resulting in the residues being changed from Ser in HZ to Trp in KLY. An SNP occurred in *ORF8*, resulting in the residues being changed from Ala in HZ to Val in KLY. White boxes indicate UTR, black boxes indicate exons, and the lines between them represent introns. (**B**) Free green fluorescent protein (GFP) and ORF3/ORF8-GFP fusion protein were transiently expressed in rice protoplasts. Green fluorescence shows GFP.

According to the Sanger sequencing results, there was a nonsynonymous mutation with T to C in exon 8 of *ORF8*, resulting in one amino acid substitution form Ala to Val. *ORF3* also had a nonsynonymous SNP with G to C in exon and caused one acid substitution from Ser to Trp. (Figure 7A). A subcellular localization analysis of *ORF3* and 8 was performed. The result showed that ORF3-GFP localized in plasma membranes and cytosol, and ORF8-GFP localized in the nucleus, plasma membranes, and cytosol (Figure 7B).

## 3. Discussion

The anther length was an essential trait in improving the outcrossing yield in rice, and the majority of QTL controlling anther size could be used in marker-assisted selection breeding. In this research, we constructed advanced backcross populations of HZ and KLY, and eight QTL sites of anther length were obtained by gene chip analysis. *qAL5.2* was fine mapped to a 73 kb region between HK139 and HK140 on chromosome 5 by seven BC_5_F_2_ populations, and candidate genes were analyzed by RNA-sequence.

In the past decade, wild rice segregating populations were used for the QTL analysis of anther size, such as *O. longistaminata* and *O. rufipogon* [13,14]. Although there were significant differences in anthers between wild rice and cultivated rice, the fine mapping of the genes remains difficult, primarily due to the challenges in phenotyping. We used two cultivated rice HZ and KLY for a QTL analysis of anther size. The significant difference in the anther/glume ratio between KLY and HZ helped to rule out the influence of the grain length QTL on anther length (Figure 1 and Figure 2). At the same time, it quickly generated the mapping populations, and we obtained seven BC_5_F_2_ populations for *qAL5.2* mapping. The genotypes of LY-1, LY-4, and LY-5 were HZ homozygous in *qAL5.2* (Figure 4), and the average anther length was 2.03 mm, 2.12 mm, and 2.15, respectively (Table S1). The anther length of LY-1 was similar to HZ; LY-4 and LY-5 were longer than HZ. We speculated that LY-4 and LY-5 populations closer to the heterozygous range were more prone to genetic recombination. The genotypes of LY-6 and LY-7 were KLY homozygous range in *qAL5.2*, named *qAL5.2^KLY^*, and the average anther length of LY-6 was 2.26 mm, which was the longest anther in all BC_5_F_2_ populations. Notably, the anther of homozygous *qAL5.2^KLY^* is not identical to KLY, indicating that other QTL may co-regulate anther size.

This research showed that the genomic regions were located on Chr1, Chr3, Chr5, Chr6, Chr8, Chr9, Chr10, and Chr12 by DNA microarray mapping (Figure 3A,B). Previously, several other groups have attempted to map QTL for anther size. Eighteen QTL for anther traits were detected using five mapping populations, and the QTL sites of Chr1, Chr3, Chr5, Chr8, and Chr9 overlapped with the current study’s locations [15]. It suggested that gene chip mapping was reliable for QTL preliminary mapping. There was no coincident QTL that was detected by all mapping populations in Uga et al., and it was similar to other research [14,16]. Therefore, we supposed that anther length was regulated by different minor QTL in different populations, and major QTL have yet to be touched. The *qAL5.2* site was close to the RM18569 marker, and a similar site was detected in multiple populations, including *O. longistaminata* (W1508), *O. rufipogon* (P16), and *indica* (IR24, T65, Aijiao Nante) [14,15,16]. *qAL5.2* should be a major QTL for anther length.

Although *qAL5* has been detected before, the candidate gene remains elusive. Based on the expression profile, there were eight predicted reading frames in the *qAL5.2* region, and only four genes showed expression in rice (Table 2). *ORF1* and *ORF3* were mainly expressed during seed development; *ORF4* and *ORF8* were mainly expressed in the panicle formation stage, and the expression of *ORF4* was also higher during the young seed stage (Table 2 and Figure S3). Only *ORF3* and *ORF8* had a nonsynonymous variation between HZ and KLY (Figure 7A), and *ORF8* encoded a lipase belonging to the alpha/beta-hydrolase (ABH) family, and have been reported in regulating plant development [17,18]. *SDP1* encodes lipase with a patatin-like acyl-hydrolase domain, mainly expressed in developing seeds, and *sdp1* exhibited a post-germinative growth arrest phenotype in Arabidopsis [19]. *RVMS* (Reversible Male Sterile) encodes a GDSL lipase/hydrolase protein predominantly expressed in anthers. The mutant of *rvms* is fertile at a low temperature (17 °C) but is male-sterile at normal temperature (24 °C), so lipase is also necessary for anther development [20,21]. And CSE, the Arabidopsis homolog protein of *ORF8*, has been reported to be involved in lignin synthesis, and *cse2* shows severe dwarfing and reduction in lignin content, so we thought *ORF8* was probably a candidate gene of *qAL5.2* [22,23].

The development of the anther is a complex process involving many regulatory pathways, such as the hormone pathway, phosphorylation pathway, and ubiquitination pathway [24,25]. In this research, we conducted an RNA-sequence experiment using an S4-stage anther of the near-isogenic line of *qAL5.2* to analyze possible regulatory pathways. The differentially expressed genes were highly enriched in the nucleic acid binding transcription and alpha-linolenic acid metabolism pathway, indicating that many enzymes or substrates related to lipid synthesis are transcribed. So we guess *qAL5.2* may be involved in regulating transcription factors and lipid metabolism in developing anthers.

In conclusion, we detected a new QTL and narrowed in a 73 kb region by fine mapping. At the same time, candidate genes of *qAL5.2* were analyzed, and RNA-sequence analysis showed that *qAL5.2* was involved in the regulation of the anther’s energy metabolism.

## 4. Materials and Methods

### 4.1. Plant Materials and Growth Conditions

*Oryza sativa* L. ssp. *indica* Huazhan (HZ) was a recurrent parent. *Oryza sativa* L. ssp. *Japonica* Poliya (PLY) was the donor parent. F1 of a big anther was identified from the HZ/KLY population derived from the cross between HZ and KLY. The BC_4_F_2_ population was derived from F1 with the recurrent parent HZ for four consecutive generations. NIL-qAL5.2^HZ^ and NIL-qAL5.2^KLY^ were derived from BC_5_F_3_. All plants were grown in the nature field at Hangzhou, Zhejiang Province of China, from May to October and at Linshui, Hainan Province of China, from January to April.

### 4.2. Measurement of Traits

The agronomic traits, including plant height, anther length, grain length, grain width and 1000-grain weight, were measured with more than three replicates at the mature stage. The anther length, anther width, grain length, and grain width were measured by SC-G software (Wanshen Detection Technology Co., Ltd., Hangzhou, China). The phenotypic variance was calculated by SPSS 17.0 software.

### 4.3. Genotype Analysis

The genomic DNA of each plant was extracted by the CTAB method [26]. For PCR amplification, a 20 µL reaction mixture consisted of 0.2 µM primers, 10 µL 2 × Taq PCR MasterMix (LSC, Hangzhou, China), and ∼15 ng of a genomic DNA sample. The PCR amplified profile was as follows: a pre-denaturation of 5 min at 94 °C, 30 cycles of 30 s at 94 °C, 30 s at 55 °C, and 30 s at 72 °C, and a final elongation at 72 °C for 5 min. Insertion and deletion markers were designed with NCBI and Primer5, and the primer sequences are listed in Table S2. The amplified products were electrophoresed in 3% agarose gel in 1× TAE buffer.

### 4.4. Genetic Mapping

The BC_4_F_2_ and BC_5_F_1_ population, individuals with extreme phenotypes of a big anther or small anther, were used for gene chip bulked segregation analysis by the company (Shuang Lv Yuang Bioinformatics Technology, Wuhan, China). Fine mapping was experimented with seven populations and nine markers by the method described previously. Briefly, genotype and phenotype analyses were conducted using Mapmaker/Exp 3.0, in which genetic distances between markers were presented in centiMorgans (cM) derived with the Kosambi function. QTL analysis was performed with the IM method by Windows QTL Cartographer 2.5, and an LOD value of 2.5 was taken as the threshold value [27].

### 4.5. Transcriptomics Analysis

About 1 g of the S4-stage (pollen microspore metaphase stage) anther of NIL-qAL5.2^HZ^ and NIL-qAL5.2^KLY^ was collected for Transcriptomics sequence. Different expression analyses, GO and KEGG, were performed by Tianjin Novogene Bioinformatics Technology. In brief, the PCR products were purified by AMPure XP system (Beckman Coulter, Pasadena, CA, USA), and the library quality was assessed using the Agilent Bioanalyzer 2100 system (Agilent, Santa Clara, CA, USA). After cluster generation, the library preparations were sequenced on the Illumina HiSeq platform (Illumina, San Diego, CA, USA) and 125 bp/150 bp paired-end reads were generated [28].

### 4.6. Subcellular Localization of qAL5

CDS of *ORF3* and *ORF8* without the stop codon were cloned into a transient expression vector PAN580-GFP to determine the subcellular localization. The fusions of GFP were transformed into protoplasts that were extracted from 15 d old HZ seedlings based on the CaCl_2_-PEG4000 method [29]. An empty PAN580-GFP vector was the control.

## Figures and Tables

**Table 1:** QTL detected for anther length in seven BC_5_F_2_ populations. A, the additive effect of replacing an HZ allele with a KLY allele; D, dominance effect; R^2^, the proportion of phenotypic variance explained by the QTL effect; ns, no significance.

Population	Heterozygous Interval	LOD	A	D	R^2^
LY-1	HK134–HK03	ns	-	-	-
LY-2	HK134–HK62	17.54	0.12	0.01	35.73
LY-3	HK03–HK62	10.19	0.08	-	25.10
LY-4	HK134–HK138	ns	-	-	-
LY-5	HK140–HK62	ns	-	-	-
LY-6	HK61–HK62	ns	-	-	-
LY-7	HK134–HK139	ns	-	-	-

**Table 2:** Candidate genes in the target region of *qAL5.2*.

ORF	ID	Gene Product	The Highest Expression
*ORF1*	LOC_Os05g29900	expressed protein	Seed-S4
*ORF2*	LOC_Os05g29910	retrotransposon protein, putative, expressed	No
*ORF3*	LOC_Os05g29920	expressed protein	Seed-S5
*ORF4*	LOC_Os05g29930	late embryogenesis abundant protein, expressed	SAM
*ORF5*	LOC_Os05g29940	expressed protein	No
*ORF6*	LOC_Os05g29950	expressed protein	No
*ORF7*	LOC_Os05g29960	expressed protein	No
*ORF8*	LOC_Os05g29974	lipase, putative, expressed	Anther

## Data Availability

The data presented in this study are available in the article or the Supplementary Materials.

## Supplementary Materials

The following supporting information can be downloaded at https://www.mdpi.com/article/10.3390/plants13081130/s1, Figure S1: Development of advanced backcross lines; Figure S2. The LOD score plots by WinQTLCart. (A) The LOD score of LY2 population. (B) The LOD score of LY3 population; Figure S3. The expression profiles of the candidate genes in different tissues; Table S1: Anther length of populations; Table S2: Prime list used in this study; Table S3: Differential expression genes list.
