# Supplementary material for: Fine Mapping of qAL5.2 Controlling Anther Length in Oryza sativa
Source: Plants (Basel). 2024 Apr 18;13(8):1130. doi: 10.3390/plants13081130 (PMC11053959; doi:10.3390/plants13081130)
Supplement: Supplementary file 1 [file plants-13-01130-s001.zip › Fine mapping of qAL5.2 controlling anther length in Oryza sativa-Supplementary Materials.pdf]

Supplemental information

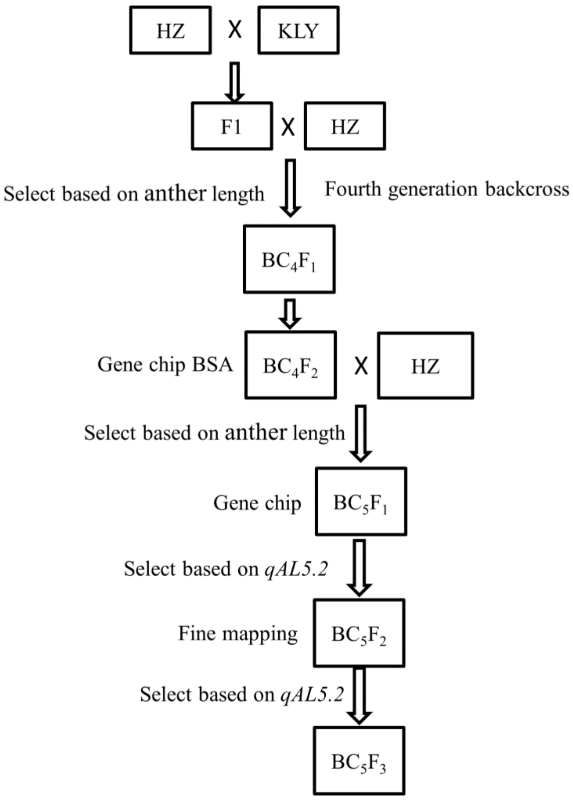

Figure S1. Development of advanced backcross lines.

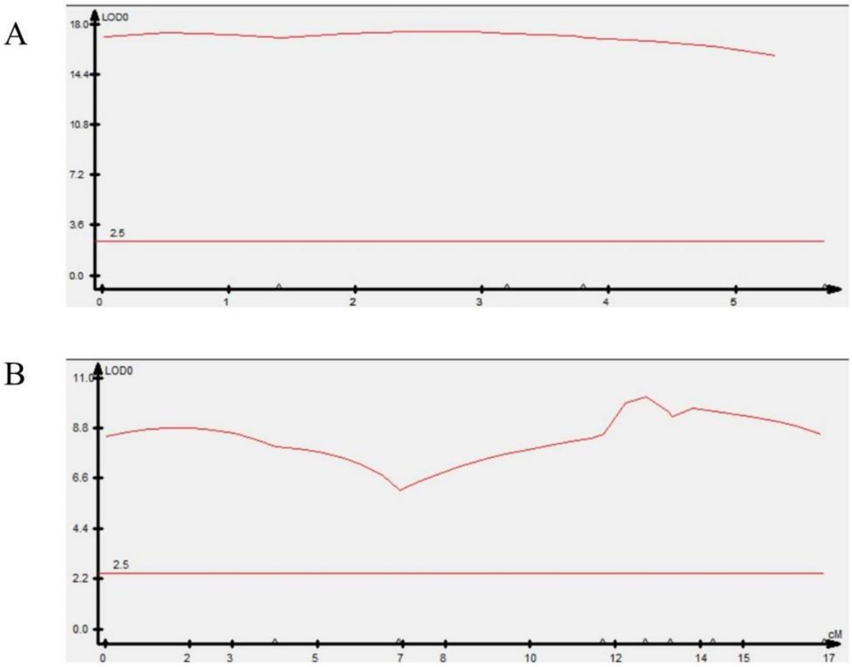

Figure S2. The LOD score plots by WinQTLCart. (A) The LOD score of LY2 population. (B) The LOD score of LY3 population.

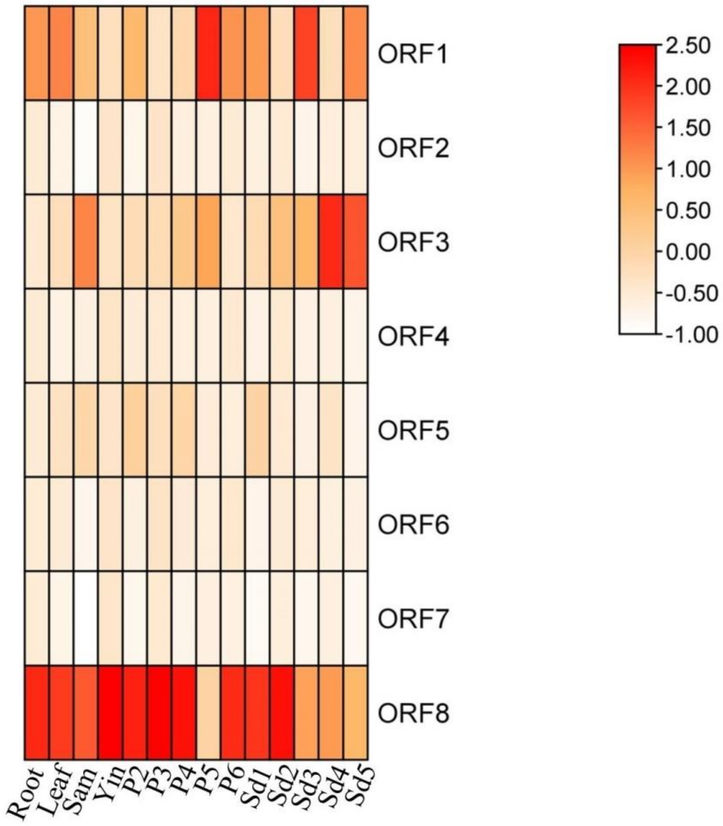

Figure S3. The expression profiles of the candidate genes in different tissues (Rice eFP Browser (utoronto.ca)).

Table S1. Anther length of populations

population	Average	Maximum	Minimum
LY-1	2.03	2.32	1.74
LY-2	2.19	2.51	1.7
LY-3	2.2	2.5	1.94
LY-4	2.12	2.33	1.85
LY-5	2.15	2.39	1.86
LY-6	2.26	2.46	2.04
LY-7	2.15	2.43	1.79

Table S2. Prime list

Prime name	Forward prime sequence (5’-3’)	Reverse prime sequence(5’-3’)
HK134	TGAAAGCCAGGAAGCATA	GAATGTGGGCAATGCTAG
HK03	TATGCCGCCACCTTGTCT	ATAGGCGGCGTTCGTGTC
HK136	AAGGATAGGCAGCGTGAA	GGCTTTGGTGGACAACTTTA
HK58	TCAGTTCCCACATAAATAAG	ACCACAATGCCCAATAGA
HK138	TGCTGGTTATCCATAGGTC	CATCGTCGTTATTAAGGGT
HK139	ATTGTGTACGACAGTTTGAATAAGT	ACAGTTGAAGGCAGGAGG
HK140	CTGTTGACTGCTGTGCCTAT	TTGGGAGTATTTGAACAGG
HK61	ATCCCTGTATCTGGGTAACGT	TTTGCCCAAACCAAGATC
HK62	GCCAAAGAAACAACGACC	CTCCAGAGCCAGCAGATT
pAN580-O	GCCCAGATCAACTAGTATGCCTCGCCTC	TCGAGACGTCTCTAGAGAACGCACCTCG
RF3	CACCTCCT	CAGTCGCA
pAN580-O	GCCCAGATCAACTAGT	TCGAGACGTCTCTAGACATCCTACATTGC
RF8	ATGACAAAGGAGATGGCCGA	TGGGGAT
seq-ORF3	GTGTTTTATTATCTTTTAATT	CACCTTGAGAACAAGAATCCGT
seq-ORF8	CAGTCCTGGTTGCGTCTC	TGGGTGGGAGCCAAAGAT
